# Normative Data and Determinants of Macular, Disc, and Peripapillary Vascular Density in Healthy Myopic Children Using Optical Coherence Tomography Angiography

**DOI:** 10.3389/fmed.2022.890294

**Published:** 2022-06-21

**Authors:** Ruru Chen, Hengli Lian, Colm McAlinden, Eirini Skiadaresi, Siyu Liu, Ting Wan, Kai Diao, Hongxian Pan, Jia Qu, Jinhai Huang, Yiyu Li

**Affiliations:** ^1^Eye Hospital and School of Ophthalmology and Optometry, Wenzhou Medical University, Wenzhou, China; ^2^Eye Institute and Department of Ophthalmology, Eye & ENT Hospital, Fudan University, Shanghai, China; ^3^NHC Key Laboratory of Myopia (Fudan University), Key Laboratory of Myopia, Chinese Academy of Medical Sciences, Shanghai, China; ^4^Department of Ophthalmology, Singleton Hospital, Swansea Bay University Health Board, Swansea, United Kingdom; ^5^Department of Ophthalmology, Royal Gwent Hospital, Aneurin Bevan University Health Board, Newport, United Kingdom; ^6^Department of Ophthalmology, Prince Philip Hospital, Hywel Dda University Health Board, Llanelli, United Kingdom; ^7^Shanghai Research Center of Ophthalmology and Optometry, Shanghai, China

**Keywords:** vascular density (VD), optical coherence tomography angiography (OCTA), macular, disc, peripapillary

## Abstract

**Objective:**

To establish a normative database for the vascular density (VD) in macular, disc, and peripapillary regions in healthy myopic children and to evaluate associated ocular features with optical coherence tomography angiography (OCTA).

**Methods:**

This was an observational, prospective and cross-sectional study. 776 Chinese healthy myopic children (375 boys and 401 girls) were enrolled, mean (±SD) age 9.84 ± 1.98 (range 6–16) years. En-face angiogram OCTA was performed on 6 mm × 6 mm retinal and 4.5 mm × 4.5 mm disc regions. VD measurements in the macular retina were segmented into the four regions: superficial capillary plexus (SCP), intermediate capillary plexus (ICP), deep capillary plexus (DCP), and choriocapillaris (CC). Correlations between macular, disc, and peripapillary VD and possible influencing factors [age, gender, axial length (AL), spherical equivalent refraction (SER), right/left eye, and signal strength index (SSI)] were assessed by Pearson’s correlation and multivariate regression analysis.

**Results:**

For macular scans, the corrected VD in the ICP region was (48.25 ± 4.24)% for the whole macular retina. The macular ICP VD in most sections was lower than the SCP, DCP, and CC (all *P* < 0.001). The corrected VD in CC was (72.96 ± 4.42)% for the whole macular retina. The macular CC VD in every section was all higher than the SCP, ICP, and DCP (all *P* < 0.001). The size of foveal avascular zone (FAZ) and foveal VD 300 (FD-300) was 0.28 mm^2^± 0.10 mm^2^ and (58.43 ± 4.17)% respectively. For disc scans, the corrected VD was (58.04 ± 2.73)% for the whole disc area. Both AL and SER were strongly correlated with ICP, DCP, and CC VD in all regions (all *P* < 0.01). Larger SSI was correlated with a lower VD in the SCP and ICP, and a higher VD in DCP and CC (all *P* < 0.01).

**Conclusion:**

Vascular density values provide large scale normative data on macular, disc, and peripapillary vascular parameters in a large sample of healthy myopic children with OCTA measured in the four different retinal plexuses and regions. The VD in different regions had various influencing factors; mainly a close correlation with AL and SSI.

## Introduction

Optical coherence tomography angiography (OCTA) is helpful in the evaluation of retinal blood flow which can be compromised in some macular or optic nerve head diseases (1, 2). Previous studies have demonstrated that OCTA has high repeatability and reproducibility in its measurements (3–5) and established the normative data (3, 5, 6) on the foveal avascular zone (FAZ) area, superficial and deep retinal vascular density (VD), disc and peripapillary VD of healthy adults.

There are some studies relating to peripapillary retinal nerve fiber layer thickness and the macular retinal thickness in children (7). However, few studies exist on the normative data of each regional retinal VD, especially the disc and peripapillary VD in healthy children. There are some differences between the eyes of children and adults. The study of adult samples is inevitably affected by age-related changes, including ocular and systemic diseases (8). Zhang et al. (9) reported that OCTA is non-invasive and reliable for evaluating macular perfusion in 8–16 year old children, but the algorithm did not apply to measurements of the disc and peripapillary VD and the sample size was small to meet the normative data measurement standards. Previous studies have assessed influencing factors such as age, gender, axial length (AL), central foveal thickness (CFT) and central foveal volume (CFV), however not comprehensively (3, 10, 11).

In this study, OCTA was used to measure the normative data on each regional superficial and deep macular, disc, and peripapillary VD in a large sample of 6–16 year old healthy Chinese children. Various ocular parameters were also measured to assess potential correlations with retinal VD.

## Materials and Methods

### Subjects and Information

This observational, prospective and cross-sectional study was conducted at the Eye Hospital of Wenzhou Medical University. A total of 714 subjects (1428 eyes) between 6 and 16 years old were recruited for this study. The inclusion criteria were as follows: (1) Uniocular corrected distance visual acuity was no worse than 20/20; (2) Intraocular pressure was between 9 and 21 mmHg; (3) Refractive error was between 0.00 and -6.00 diopters (D); (4) No history of systemic disease or any evidence of retinal pathology. This study was approved by the Medical Ethics Committee of the Eye Hospital of Wenzhou Medical University. Written informed consent was obtained from parents of participants. Besides, the poor OCTA image quality was evaluated by the low signal strength index (SSI) and the low quality index (QI), which reflects the signal strength, motion artifacts and image sharpness respectively. Therefore, subjects with poor quality images on OCTA (SSI lower than 55 and/or QI lower than 7) were excluded. We also excluded blink or doubling artifacts, and/or segmentation errors. All subjects underwent standard ophthalmic examinations, cycloplegic autorefraction (Nidek, Gamagori, Japan), axial length (IOL-Master 500, Carl Zeiss, Germany), and OCTA imaging in both eyes (RTVue XR Avanti; Optovue, Inc., Fremont, CA, United States). All measurements and OCTA images were performed by the same skilled examiner (SHM). All OCTA images were checked by one expert for quality and correctness of automated layer segmentation.

### Optical Coherence Tomography Angiography and Parameters

The VD measurements of macula, disc, and peripapillary regions were generated by OCTA using the RTVue XR Avanti device with the AngioVue Analytics software (version 2018.0.0.14) to analyze image. This software includes the 3D PAR algorithm, which could remove projection artifacts of image. The Angio Retina HD mode (6 mm × 6 mm) and the Disc (4.5 mm × 4.5 mm) mode were performed on each eye with horizontal and vertical orthogonal OCTA volume scans at the fixed position. The OCTA (12) showed the high repeatability in vascular parameters of 6 mm × 6 mm macular area and the 4.5 mm × 4.5 mm peripapillary area on healthy children. 6 mm × 6 mm macular mode was widely used in recent studies (11, 12). The measurement principle of angiography and flow images has been introduced in previous studies (3, 13).

The macular VD (VD) was defined as the ratio of the area of the large vessel and capillary vessel divided by the total area measured in particular sections. The VD of the fovea, parafovea and perifovea were acquired through 6 mm × 6 mm HD mode OCTA images were calculated by AngioVue Analytics software. In accordance with the “Early Treatment Diabetic Retinopathy Study” (ETDRS) subsegment method, the parafovea and perifovea regions were divided into four quadrants, namely superior, inferior, temporal, and nasal, respectively, and two equal hemispheres (superior and inferior). In addition, retinal OCT angiographs was automatically layered into superficial vascular plexus (SVP) and the deep vascular complex (DVC) by the standard commercial software traditionally (14), which made intermediate and deep capillary plexus (DCP) was disturbed by projection artifacts originating by the superficial capillary plexus (SCP) (14, 15). Therefore, we segmented en face OCT angiographs manually into the four segmentations by adjusting the value of layer boundaries in the custom segmentation software. (1) SVP was set between the internal limiting membrane (ILM) and 9 μm above the junction between the inner plexiform layer and the inner nuclear layer (IPL–INL); (2) the intermediate capillary plexus (ICP) was comprised between 9 μm above the IPL–INL junction and 6 μm below the inner plexiform layer and the outer plexiform layer (INL–OPL) junction; (3) the DCP extended from 6 μm below the INL– OPL junction to 9 μm below OPL and outer nuclear layer (OPL–ONL) junction (16). The validation of the custom segmentation method was confirmed by most recent researches; (12, 14, 16) (4) choriocapillaris (CC) was set as the distance between 9 μm from the Bruch membrane (BM) and 31 μm below automatically segmented by the software. The VD was automatically calculated by the AngioVue Analytics software.

For 4.5 mm × 4.5 mm optic disc scan, parameters of the optic nerve head (ONH) VD referred to the whole VD (including all vessel) and whole capillary VD (only including capillary). The whole capillary VD was divided into the inside disc capillary VD referring to the area within the optic disc boundary and the peripapillary VD that was defined as a 750-mm-wide elliptical annulus extending from the optic disc boundary. The VD of disc or peripapillary capillary were measured from the ILM to the posterior boundary of the retinal nerve fiber layer (RNFL). RNFL thickness and the peripapillary thickness also were measured in this study. According to the Garway Heath Partition Method, the peripapillary area was subdivided into eight sections including nasal superior, nasal inferior, inferior nasal, inferior temporal, temporal inferior, temporal superior, superior temporal, and superior nasal.

**FIGURE 1:**
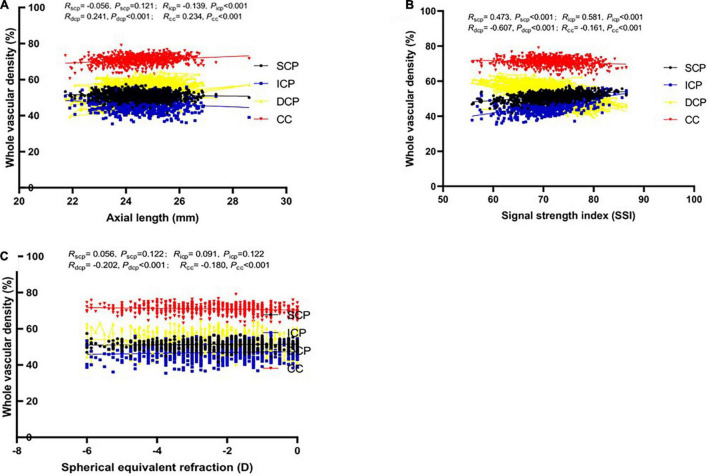
Relationship between vascular density (VD) in whole macular retina and axial length (mm) in four different vascular plexuses (SCP: superficial capillary plexus; ICP: intermediate capillary plexus; DCP: deep capillary plexus; CC: choriocapillaris). Boxes and lines of black, blue, yellow and red color depict the relationship between the possible influencing factors [**(A)** Axial length; **(B)** Signal strength index; **(C)** Spherical equivalent refraction] and VD in SVP, DVC, ICP, and DCP, respectively.

With image scan, the area of FAZ was automatically in mm^2^ outlined and calculated by this software using the FAZ measure function. Perimeter and acicularity Index of FAZ also was calculated. The macular, disc, and peripapillary vasculature was automatically segmented by the software (shown in Figure 1). Foveal VD 300 (FD-300) was defined as VD in a 300-mm wide zone around the FAZ combining the SVP and the DVC. Area density and length density of FD-300 automatically calculated by the software and analyzed in this study. CFT and central foveal volume were measured automatically as the central 1 mm foveal thickness or volume among all retinal layers with the caliper tool in the OCT software.

### Optical Coherence Tomography Angiography Image Magnification Correction

Theoretically, the ocular magnification of an OCTA image is equal to 1 when a default axial length of 23.82 mm is adopted by the RTVue XR Avanti system. In order to obtain the corrected or true OCTA images data, original OCTA images were adjusted by axial length by the Littman and the modified Bennett formulae (17). The relationship between the original OCTA image diameter (Dm) and the true diameter (Dt) can be expressed as: Dt = p × q × Dm, where p × q is the overall image magnification factor. The factor q can be determined by the Bennett formula: *q* = 0.01306 × (AL – 1.82), p can be calculated as 1/[0.01306 × (23.824.46 – 1.82)] = 3.48 according to RTVue XR Avanti system (18). Therefore, the magnification factor of the image should be corrected by: Dt/Dm = p × q = 3.48 × 0.01306 × (AL – 1.82). This formula was utilized to correct the en face OCTA image.

## Statistical Analysis

Statistical analysis was performed using SPSS statistical software (V25.0, IBM Corp.). A sample Kolmogorov–Smirnov (K–S) test was used to verify the normality of the factors such as age, AL, spherical equivalent refraction (SER), and VD in each section of the macula, disc, and peripapillary parameters. Pearson’s correlation was used to assess correlation with possible influencing factors and VD parameters. The correlation between different plexuses in macular, disc, and peripapillary VD was also analyzed by Pearson’s correlation. We conducted a multivariate stepwise linear regression analysis to identify the main factors determining a greater or lesser macular and disc VD. The children normative VD values in macular and disc were corrected by the magnification factor and were reported as the mean with standard deviation and the range from minimum values and maximum values. The significance value was set at 5%.

## Results

### Demographic Results

In total, 793 healthy children were enrolled in this study. Fourteen children were excluded due to poor quality images with macular (10 children) and optic disc (6 children) scans and segmentation errors (1 child). Therefore, the study included 1,552 eyes of 776 Chinese children (375 boys and 401 girls) with a mean (± SD) age of 9.84 ± 1.98 (range 6–16) years. The characteristics of the eyes, whole macular VD in different layers and disc retina VD in the children studied were shown in Table 1.

**TABLE 1:** Characteristics of the eyes, macular VD, and disc VD in the children studied.

Parameters	Mean ± standard deviation	Range
Age (year)	9.84 ± 1.98	6 – 16
Gender (boys: girls)	375: 401	–
Axial length (mm)	24.50 ± 0.96	21.73 – 28.61
SER (D)	−2.16 ± 1.44	−6.00 – 0.00
Astigmatism (D)	−0.51 ± 0.54	−3.50 – 0.00
Macular SSI	71.86 ± 5.43	55.78 – 86.49
Overall quality index of macula	8.34 ± 0.60	7.00 – 9.00
Disc SSI	74.89 ± 7.53	55.01 – 91.12
Overall quality index of disc	8.56 ± 0.60	7.00 – 10.00
CFT	241.16 ± 18.02	187.80 – 378.20
CFV	0.19 ± 0.01	0.15 – 0.30
TS	89.77 ± 13.34	41.10 – 164.40
Disc area (mm^2^)	2.25 ± 0.46	1.00 – 4.60

*VD, vascular density; SSI, signal strength index; SCP: superficial capillary plexus; ICP: intermediate capillary plexus; DCP: deep capillary plexus; CC: choriocapillaris; CFT: central foveal thickness; CFV: central foveal volume; TS, total thickness of never fiber; SER, spherical equivalent refraction.*

### Correlation Between Macular Vascular Density Parameters and the Possible Influencing Factors

**FIGURE 2:**
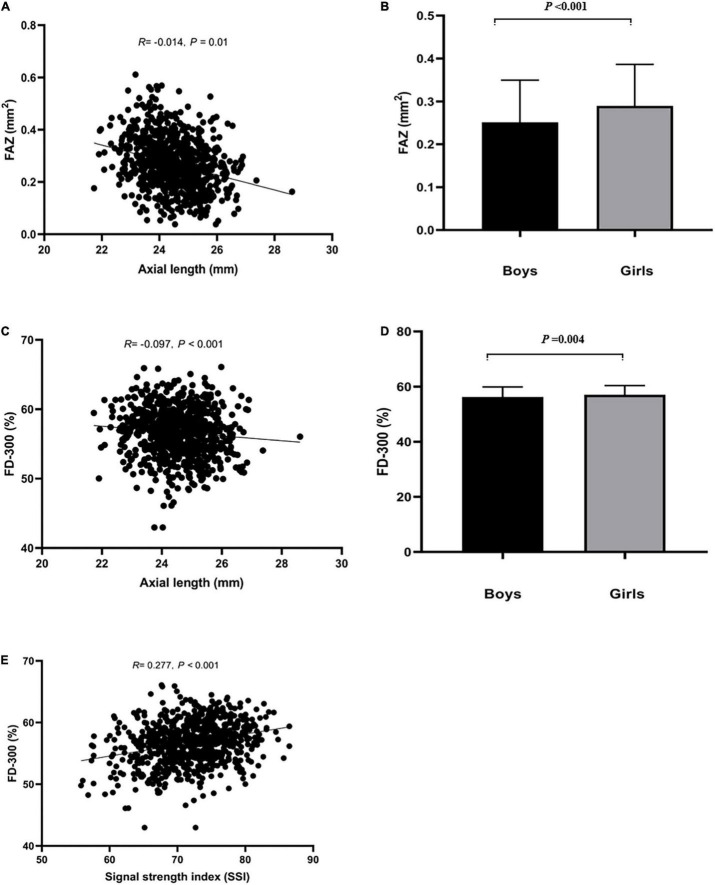
Relationship between FAZ, FD-300 and axial length (mm), SSI, and sex. **(A,B)** Represented that axial length and sex influenced FAZ. **(C–E)** Represented that axial length, SSI and sex influenced FD-300.

**FIGURE 3:**
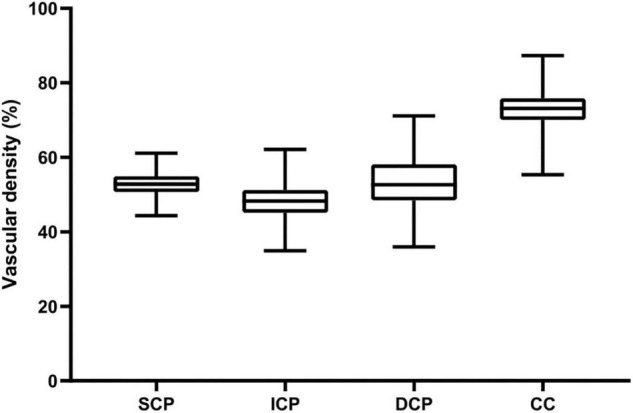
Vascular density of the four retinal vascular plexuses in children. Box-plots show whole VD values within the SVP, DVC, ICP, and DCP, respectively. Vascular density (%) is represented on the *y*-axis. Upper and lower whiskers represent the upper and lower values. Upper and lower box margins represent the 25th and 75th percentiles. The white line inside the box is the median value. The VD is significantly different between each plexus (all *P* < 0.001), higher in the CC, followed by the DVP, the SCP, and the ICP.

Supplementary Table 1 demonstrate the associations between macular VD and gender, age, AL, SER, SSI, CFT, CFV, eye. Gender was revealed to be positively correlated with the ICP VD in most macular regions, only negatively in fovea region (*P* < 0.001), correlated with the SCP VD only in three regions (*P* < 0.05), and not correlated with the DCP VD or CC VD in any region (*P* > 0.05). Age was significantly positively correlated with DCP VD and CC VD in all macular region (*P* < 0.01), not correlated with SCP or ICP VD in most regions (*P* > 0.05). Both AL and SER, were strongly correlated with ICP, DCP, and CC VD in all regions (*P* < 0.01), and related with SCP VD in only three regions (*P* < 0.05). A longer AL or lower refraction could imply larger ICP VD and lower DCP and CC VD (Figures 1, 2). SSI was significantly correlated with all macular VD with different layers in all regions (*P* < 0.01) (Figure 3). Larger SSI led to a lower VD of SCP and ICP and a larger VD of DCP and CC. CFT or CFV was almost not correlated with VD with four layers in all regions (*P* > 0.05), except for superior, nasal parafovea in SCP, superior parafovea in ICP, and fovea in DCP (*P* < 0.05). The VD of right eyes in SCP, ICP, and CC was closely related to those of the left eyes (*P* < 0.01) but DCP VD.

FAZ area, FAZ perimeter, FD-300 area and FD-300 length was larger in girls than boys (all *P* < 0.05). There was close relationship in those parameters between right and left eyes (*P* < 0.05). A shorter AL implied a smaller FAZ and FD-300 area (*P* < 0.05), and higher SSI implied a larger FD-300 area and FD-300 length (*P* < 0.05). Those parameters was not associated with age, SER, CFT, and CFV (*P* > 0.05), as shown in Supplementary Table 1.

In multivariate regression analysis (Table 2), only SSI remained as a determinant of SCP VD, ICP VD and FD-300 area (*P* < 0.05). AL and SSI persisted as significant determinants of DCP VD and CC VD (*P* < 0.05). A larger FAZ area was mainly found in girls and determined by a shorted AL (*P* < 0.05).

**TABLE 2:** Multivariate regression analysis of factors determining a greater or lesser whole macular, inside disc, and peripapillary vascular density.

Dependent variable	SCP		ICP		DCP		CC		FAZ area		FD-300	
						
	B	P	B	P	B	P	B	P	B	P	B	P
Constant	35.751	0.000	19.534	0.000	70.379	0.000	62.434	0.000	0.873**	0.000	49.927**	0.000
Gender	0.144	0.361	0.455	0.082	0.609	0.077	0.068	0.727	0.023**	0.004	0.503	0.073
Age	0.001	0.999	0.081	0.232	–0.138	0.12	0.073	0.143	0.003	0.118	0.002	0.98
AL	0.070	0.521	–0.243	0.177	0.947**	0.000	0.401**	0.003	−0.028**	0.000	–0.134	0.487
SER	–0.088	0.200	–0.163	0.152	0.025	0.869	–0.079	0.352	–0.002	0.567	–0.116	0.342
SSI	0.192**	0.000	0.421**	0.000	−0.579**	0.000	−0.056**	0.002	0.001	0.174	0.156**	0.000
CFT	0.345	0.082	0.243	0.461	0.152	0.726	–0.198	0.417	0.010	0.328	0.415	0.239
CFV	–439.07	0.081	–301.89	0.469	–191.64	0.727	259.389	0.401	–12.539	0.313	–537.68	0.228

**Dependent variable**	**Inside disc**						**Peripapillary**					
		
	**B**			**P**			B			**P**		

Constant	44.46			0.000			63.375			0.000		
Gender	0.398*			0.044			0.468**			0.003		
Age	0.116*			0.029			−0.032			0.458		
AL	0.173			0.202			−0.279**			0.01		
SER	−0.216*			0.014			0.106			0.132		
SSI	0.146**			0.000			−0.027**			0.009		
Disc area	0.001			0.845			0.013*			0.011		
TS	0.687**			0.001			0.923**			0.000		

B, Unstandarized beta; *P < 0.05 and **P < 0.01 were marked as red color. SCP, superficial capillary plexus; ICP, intermediate capillary plexus; DCP, deep capillary plexus; CC, choriocapillaris. AL, axial length; SER, spherical equivalent refraction; SSI, signal strength index; CFT, central foveal thickness; CFV, central foveal volume.

### Correlation Between Vascular Density in Disc and Peripapillary Region and the Possible Influencing Factors

At the disc and peripapillary region (Supplementary Table 2), the whole VD was higher in girls than boys, with a smaller age, shorter AL, greater SER, larger disc area, and larger TS (*P* < 0.05). The inside disc VD was also higher in girls than boys, with a higher SSI and a larger disc area (*P* < 0.05). The peripapillary VD was higher in girls than boys (*P* < 0.05), with a shorter AL, larger SER, larger disc area, and larger TS (*P* < 0.05). There was close relationship in those parameters between right and left eyes (*P* < 0.05). The relationship between the possible factors and each regional peripapillary and disc VD is detailed in Table 3. In lineal regression analysis (Table 4), gender, age, SER, SSI, and TS all remained as determinants of inside disc VD (*P* < 0.05). Gender, AL, SSI, disc area, and TS persisted as significant determinants of peripapillary VD (*P* < 0.05).

**TABLE 3:** Correlations between whole macular VD measured in the different vascular plexuses and disc VD.

Parameters		Correlation	*P*-value
SCP -	ICP	0.394**	0.000
	DCP	−0.446**	0.000
	CC	−0.122**	0.001
	Whole disc	0.280**	0.000
	Inside disc	0.186**	0.000
	Peripapillary	0.213**	0.000
ICP -	DCP	−0.628**	0.000
	CC	0.057	0.114
	Whole disc	0.087*	0.015
	Inside disc	0.187**	0.000
	Peripapillary	0.040	0.265
DCP -	CC	0.075*	0.037
	Whole disc	−0.117**	0.001
	Inside disc	−0.151**	0.000
	Peripapillary	–0.017	0.636
CC -	Whole disc	–0.070	0.050
	Inside disc	0.040	0.270
	Peripapillary	–0.020	0.571
Whole disc -	Inside disc	0.371**	0.000
	Peripapillary	0.910**	0.000
Inside disc-	Peripapillary	0.216**	0.000

VD, vascular density; SCP, superficial capillary plexus; ICP, intermediate capillary plexus; DCP, deep capillary plexus; CC, choriocapillaris. *P < 0.05 and **P < 0.01.

**TABLE 4:** The corrected macular vascular density of normal eyes in children with 6 mm × 6-mm scan pattern [Mean ± standard deviation (range)].

Section	SCP	ICP	DCP	CC	*P*-value
Whole retina	52.86 ± 3.08 (44.32 – 61.11)	48.25 ± 4.24 (34.91 – 62.11)	53.30 ± 6.43 (35.95 – 71.14)	72.96 ± 4.42 (55.34 – 87.31)	<0.001
Superior-hemi	53.19 ± 3.11 (45.33 – 61.79)	49.00 ± 4.37 (35.90 – 62.63)	52.82 ± 6.27 (35.85 – 71.95)	73.22 ± 4.50 (54.62 – 88.41)	<0.001
Inferior-hemi	52.50 ± 3.18 (42.11 – 61.32)	47.47 ± 4.32 (33.78 – 61.54)	53.78 ± 6.85 (36.05 – 70.70)	72.69 ± 4.47 (56.08 – 86.22)	<0.001
Fovea	23.90 ± 6.93 (4.93 – 47.52)	40.23 ± 7.60 (18.50 – 62.70)	33.52 ± 8.57 (11.04 – 63.84)	75.70 ± 5.19 (51.47 – 92.40)	<0.001
Parafovea	55.82 ± 3.64 (41.39 – 66.59)	51.15 ± 3.70 (38.98 – 62.80)	53.58 ± 6.40 (36.92 – 73.50)	70.31 ± 5.11 (52.07 – 84.34)	<0.001
-Superior-hemi	56.21 ± 3.63 (43.29 – 67.85)	51.45 ± 3. 84 (38.96 – 63.89)	53.26 ± 5. 88 (38.07 – 73.54)	70.00 ± 5.26 (50.56 – 83.55)	<0.001
-Inferior-hemi	55.43 ± 3.90 (39.24 – 66.8)	50.85 ± 3.75 (37.48 – 61.85)	53.91 ± 7.19 (35.78 – 74.84)	70.61 ± 5.16 (53.59 – 85.14)	<0.001
-Temporal	55.89 ± 3.63 (41.88 – 68.35)	52.62 ± 3.89 (37.82 – 66.10)	53.79 ± 6.57 (34.94 – 74.92)	72.07 ± 5.23 (53.70 – 87.08)	<0.001
-Superior	56.73 ± 4.01 (39.47 – 68.53)	50.12 ± 4.13 (35.77 – 62.15)	52.85 ± 5.96 (36.78 – 72.96)	52.85 ± 5.50 (51.16 – 83.32)	<0.001
-Nasal	55.02 ± 3.91 (38.82 – 67.96)	52.37 ± 3.83 (38.83 – 65.56)	53.81 ± 6.66 (37.67 – 76.36)	70.87 ± 5.44 (51.38 – 86.18)	<0.001
-Inferior	55.62 ± 4.33 (40.35 – 66.89)	49.49 ± 4.15 (37.33 – 61.24)	53.89 ± 7.65 (35.88 – 75.26)	69.70 ± 5.51 (50.46 – 85.64)	<0.001
Perifovea	53.58 ± 3.20 (43.65 – 62.36)	48.95 ± 4.49 (34.92 – 64.34)	54.13 ± 6.87 (35.92 – 74.81)	73.64 ± 4.53 (55.46 – 88.27)	<0.001
-Superior-hemi	53.89 ± 3.20 (45.40 – 62.84)	49.70 ± 4.52 (36.35 – 65.22)	53.33 ± 6.54 (35.51 – 75.23)	73.68 ± 4.58 (54.74 – 88.76)	<0.001
-Inferior-hemi	53.26 ± 3.36 (41.85 – 62.44)	48.19 ± 4.70 (32.67 – 63.46)	54.94 ± 7.57 (36.33 – 74.39)	73.59 ± 4.60 (56.19 – 87.79)	<0.001
-Temporal	49.54 ± 3.30 (37.81 – 59.49)	50.59 ± 3.84 (37.81 – 63.78)	55.05 ± 6.46 (37.81 – 74.16)	74.94 ± 4.69 (57.32 – 89.49)	<0.001
-Superior	54.00 ± 3.37 (44.38 – 63.78)	49.54 ± 4.96 (32.04 – 66.44)	53.21 ± 6.79 (36.39 – 74.74)	73.53 ± 4.79 (54.42 – 89.98)	<0.001
-Nasal	57.45 ± 3.54 (46.36 – 69.46)	48.15 ± 5.21 (33.75 – 64.33)	52.77 ± 7.60 (34.12 – 76.93)	72.58 ± 4.39 (55.09 – 85.60)	<0.001
-Inferior	53.39 ± 3.64 (41.35 – 63.42)	47.48 ± 5.25 (31.28 – 62.78)	55.48 ± 8.10 (36.19 – 74.34)	73.47 ± 4.92 (55.02 – 87.93)	<0.001

*SCP, superficial capillary plexus; ICP, intermediate capillary plexus; DCP, deep capillary plexus; CC, choriocapillaris.*

### Correlation Between Macular Vascular Density in Different Layers and Vascular Density in Disc and Peripapillary Region

There were close relationships between whole macular VD in any two layers, except for no relationship between ICP and CC VD (*P* > 0.05). A larger SCP VD related to a larger ICP VD, a lower DCP VD (*P* < 0.05), and a lower CC VD. A larger DCP VD implied a lower ICP VD and a larger CC VD (*P* < 0.05). There were positive relationships between whole disc VD, inside disc VD, and peripapillary VD (*P* < 0.05). SCP VD was positively correlated with whole disc VD, inside disc VD, and peripapillary VD (*P* < 0.05). CC VD was not corrected with those VD in disc. ICP VD and DCP VD were correlated with whole disc VD and inside disc VD (*P* < 0.05). The details were showed in Table 3.

### The Corrected Optical Coherence Tomography Angiography Parameters

For macular scans, the SCP VD were (52.86 ± 3.08)%, (23.90 ± 6.93)%, (55.82 ± 3.64)%, and (53.58 ± 3.20)% for the whole, fovea, parafovea, and perifovea respectively. The ICP VD were (48.25 ± 4.24)%, (40.23 ± 7.60)%, (51.15 ± 3.70)%, and (48.95 ± 4.49)% for the whole, fovea, parafovea, and perifovea respectively. The ICP VD for every section was the lowest among SCP, ICP, DCP, and CC in macular retina (all ***P*** < 0.001) (Figure 3), except for VD in fovea. The DCP VD were (53.30 ± 6.43)%, (33.52 ± 8.57)%, (53.58 ± 6.40)%, and (54.13 ± 6.87)% for the whole, fovea, parafovea, and perifovea respectively. The CC VD were (72.96 ± 4.42)%, (75.70 ± 5.19)%, (70.31 ± 5.11)%, and (73.64 ± 4.53)% for the whole, fovea, parafovea, and perifovea respectively. The CC VD in every section was the highest among four subsegments in macular retina (all ***P*** < 0.001). The highest VD in CC was in the fovea among different area. The size of foveal FAZ area and perimeter was 0.28 ***mm***^2^ ± 0.10 mm^2^ and 2.02 ***mm*** ± 0.38 mm. The density of FD-300 area was (58.43 ± 4.17)%. The normative data of macular VD in the four subsegments were shown in Tables 4, 5.

**TABLE 5:** The corrected parameters of FAZ and FD-300 of normal eyes in children with 6 mm × 6-mm scan pattern [Mean ± standard deviation (range)].

Parameters	Mean ± SD (range)
FAZ area	0.28 ± 0.10 (0.04 – 0.59)
FAZ perimeter	2.02 ± 0.38 (0.76 – 3.08)
FAZ acircularity index	1.12 ± 0.06 (0.97 – 1.31)
FD-300 area density	58.43 ± 4.17 (42.82 – 72.56)
FD-300 length density	13.93 ± 1.10 (8.92 – 17.05)

For disc and peripapillary scans, VD vs. capillary VD was (58.04 ± 2.73)% vs. (51.02 ± 2.69)%, (65.41 ± 4.10)% vs. (56.69 ± 4.95)%, and (59.68 ± 3.08)% vs. (52.58 ± 3.14)% for the whole, disc, and peripapillary. The VD in temporal inferior peripapillary was significantly higher that other section of peripapillary (*P* < 0.001). The normative data of peripapillary VD in the subsegments were shown in Table 6.

**TABLE 6:** The corrected disc and peripapillary vascular density of normal eyes in children with 4.5 mm × 4.5-mm scan pattern. [Mean ± standard deviation (range)].

Section	Vascular density (%)
Whole	58.04 ± 2.73 (48.34 – 68.71)
Whole capillary	51.02 ± 2.69 (41.94 – 61.61)
Inside disc	65.41 ± 4.10 (49.79 – 77.84)
Inside disc capillary	56.69 ± 4.95 (41.02 – 70.72)
Peripapillary	59.68 ± 3.08 (50.86 – 71.08)
Peripapillary capillary	52.58 ± 3.14 (43.18 – 64.23)
-Superior hemi	60.00 ± 3.20 (50.25 – 72.14)
-Superior hemi capillary	59.33 ± 3.16 (50.84 – 70.77)
-Inferior hemi	52.62 ± 3.37 (42.96 – 65.25)
-Inferior hemi capillary	52.53 ± 3.28 (42.52 – 64.39)
-Nasal superior	48.73 ± 4.05 (34.00 – 61.40)
-Nasal inferior	47.90 ± 4.47 (31.10 – 62.94)
-Inferior nasal	50.13 ± 4.27 (36.10 – 64.38)
-Inferior temporal	57.17 ± 4.51 (44.91 – 72.28)
-Temporal superior	56.65 ± 4.40 (43.97 – 71.58)
-Temporal inferior	57.72 ± 3.96 (45.53 – 70.92)
-Superior temporal	55.51 ± 4.36 (41.11 – 67.50)
-Superior nasal	49.80 ± 4.61 (31.50 – 66.79)

## Discussion

Optical coherence tomography angiography is an excellent resource to assess retinal microvasculature within the macular and peripapillary regions in children (19). Myopia has become one of the leading causes of visual impairment worldwide. Recently, the more and more attention has been paid to this close relationship between myopia and vessel density in the macular and peripapillary retina. This study aimed to provide large scale normative data of macular, disc, and peripapillary VD measurements in a large number of healthy myopic children, and to evaluate the effects of gender, age, axial length, refraction, and SSI on vessel density for macular, disc, and peripapillary VD.

The enlargement of the FAZ is a sign associated with retinal microvascular disease. The size of the FAZ area in children of 6–16 years old was 0.28 mm^2^ (perimeter of 2.02 mm and acicularity index of 1.12) in the present study. The result was similar to that reported by Zhang et al. (9) and Hasan et al. (11). In their studies, at a mean value of 0.29 and 0.28 mm^2^ in 6–16 year old children were reported, respectively. The perimeter of FAZ and acicularity index in adults is also in line with our studies, 1.99 mm and 1.08, respectively (18). The FAZ in children was within the range of the mean FAZ in the healthy adult, ranging from 0.25 to 0.47 mm. (2, 3, 20, 21). However, these results were different from the results in 4–15 year old children reported by Li et al. (22) ranging from 0.22 to 0.25 mm^2^. While the correlation analyzes (Supplementary Table 1) found girls had significantly larger FAZ area than boys and VD at the fovea in the SCP and DCP was significantly greater in boys. This close relationship between FAZ size and sex were greatly consistent with the children studies by İçel et al. (23) and Kiziltoprak et al. (11). The difference in the area and perimeter of FAZ values between different studies may be due to this sex ratio, in addition to different devices, measurement methods, algorithm version, sample size and ethnicity. However, there are some studies in adults with opposite findings (24, 25), which may be caused by the difference in age. In previous studies in adults, the effects of refraction and AL should be considered in the measurement of FAZ size (26) and age, CFT, and CFV may also affect the size of the FAZ (9, 11, 24). However, these factors did not affect the size and perimeter of FAZ in this large sample study. Hasan et al. (11) also had a similar result with the current results. Thus, the effects of oxygen supply to the retina and individual differences in foveal shape on the FAZ size require further investigation.

The largest VD was observed in the CC among the four different vascular plexuses in macular retina in all sections of the macular vascular layers. Table 4 and Figure 3 showed the CC VD was 72.96 and 70.31% in the whole and parafovea of retina, respectively, which were greatly higher than VD in the SCP, ICP, and DCP (48.25 to 53.30% and 51.15 to 55.82%, respectively). Seung et al. (24) also found the similar advantage in CC compared to SCP and DCP. In any sections of macular vascular layers, we considered that choriocapillaris played a more major role in supplying blood to the retina. The higher CC VD was found in fovea than CC VD in other region, which may be explained by the fact that the FAZ exists in the retinal vasculature and there is only CC that carries blood to photoreceptors. In this study, the smallest VD were found in ICP, only except for VD in fovea. Differences in VD between four different subsegments in macular retina have also been reported in healthy adult eyes (3, 27). The correlation between whole macular VD measured in the different vascular plexuses (Table 3) showed that VD of the four vascular plexuses had a close relationship between each other, except that no correlation between ICP and DCP. The value of superficial VD increased with intermediate VD, and decreased with deep VD and choriocapillaris VD. The value of deep VD was positively related to choriocapillaris VD. Lavia et al. (28) also found the similar strong positively correlation between SCP and ICP in adults, while they also found the positive relationship between SCP and DCP and that ICP and DCP were correlated with each other, which was different with the current study. However, it was lack of OCTA studies with myopic children recently. Thus, this difference may be explained by the various subject population.

The correlation (Supplementary Table 2) between one retinal plexuses and the potential influence factors revealed that gender mainly influenced SCP and ICP VD. In the most sections of ICP and DCP, girls had higher VD than boy in the current study, but lower VD in fovea retina. Conversely, in healthy adults’ eyes, it was also revealed that superficial VD was higher in men than women (10), which may be explained that males have thicker macula than females (29). Currently, the reason about regional gender differences in retinal macular VD are not known. In this study, we found age mainly influenced DCP VD and CC VD. DCP and CC VD in all section of macular increased with increasing age and the same results were observed in other studies (2, 5, 8). However, Yu et al. (30) hold an opposite view that lower superficial retinal small vessel network density was associated with older age, observed in adults. There was a regional difference in the relationship between age and macular VD. The specific mechanism remains to be further studied. Both the correlation analysis and multivariate analysis revealed that VD in four subsegments layers all were influenced by SSI, and that the longer baseline AL led to a higher VD in the DCP and CC. The most previous studies in adults also revealed the similar correlation that AL (18) had a significant effect on the value of VD in any plexuses. Therefore, in this study, original OCTA images were adjusted by axial length by the Littman and the modified Bennett formulae (the magnification factor) (17). Our study confirmed that the higher the SSI, the higher the VD in the macula, disc regions, which was consistent with previous studies (8, 9). The VD of SCP, ICP, and CC in all section of macular retina were the same in the right and left eyes consistent with the most of previous studies (5, 6, 31), However, interestingly, DCP VD of right eyes was different from DCP VD of left eyes. However, no relationship between SER, and VD was found in this study with a larger number of data, which was consistent with the study by Zhang et al. (9) and Wang et al. (10) Despite macular VD being lower in thinner center macular thickness (CFT) (11, 32), we got a negative result that there was no correlation between macular VD and either CFT or CFV, which was similar with the study by Wang et al. (10) and Pinhas et al. (33) found increased superficial microvascular densities along with thicker inner retinal thickness without outer retinal thickness. There was a report that CFT was only related to the VD of DCP rather than the VD of SCP and choroid (6). This was consistent with the known conclusion that the retina has two sets of blood supply systems (34).

This is the first study to measure disc and peripapillary VD with and without large vessels in large sample size of children by RTVue-XR Avanti algorithm. The mean disc and peripapillary VD were 65.41 and 59.68%, and reduced by about 7% after the subtraction of large vessels. The VD in the temporal peripapillary region was greater than nasally, which was similar with findings in adults (10, 31). The whole VD mainly in the optic disc was greater in girls than boys. A study found no gender differences in the whole and peripapillary VD and the VD was greater in males than females only in the temporal and inferotemporal peripapillary area. Differences may come from different subsegment methods, algorithms and sample sizes. The reports about the effect of gender on disc and peripapillary VD are sparse and the reason is unclear and requires further research. Our study found that disc and peripapillary VD had no difference in the left and right eyes. Childhood age affected the whole VD, the authors hold a view that the disc VD increased with age in childhood and may decrease after 30 years old (35), while the density around the disc was opposite and may remain unchanged during adulthood after 30 years old (35). To confirm this conclusion, longitudinal follow-up is needed.

This study was a large sample prospective study to report normative data of VD and analyze various clinical and demographic factors that may affect VD measurement. However, this study had some limitations. First, the 6 × 6 not 3 × 3 macular scan pattern was chosen in this study. The regional perifoveal VD based on the 6 × 6 scan pattern was first reported in this study. If two models were included at the same time, there was concern about visual fatigue in children. Second, subjects in this study was limited to the Chinese myopic children. More children coming from different countries and emmetropic eyes were needed to study in future. Third, choroidal thickness was not included in the study because there is no method for objectively measuring this parameter currently. Fourth, due to the limitations of the algorithm, it was not possible to analyze the normative data and influencing factors of the VD in subsegments from choroid and outer retina. Finally, longitudinal studies are needed to observe the effect of age on various regions of retinal VD.

These findings are a baseline reference providing normative data on the size of the FAZ, FD-300 and VD at the macula, disc, and peripapillary regions in a large sample of healthy myopic children in China with the RTVue XR-Optovue OCTA. We also describe how retinal perfusion parameters are influenced in each individual region by gender, age, eye, AL, SER, SSI, CFT, CFV, total thickness of never fiber and disc area. These data in a large sample can be used in future studies and provide a reference for the development of blood flow diseases involving the macula or ONH.

## Data Availability Statement

The original contributions presented in this study are included in the article/Supplementary Material, further inquiries can be directed to the corresponding authors.

## Ethics Statement

The studies involving human participants were reviewed and approved by the Medical Ethics Committee of the Eye Hospital of Wenzhou Medical University. Written informed consent to participate in this study was provided by the participants’ legal guardian/next of kin.

## Author Contributions

RC, HL, JQ, JH, and YL contributed to the conception and design of the study. SL, TW, KD, and HP organized the database. HL performed the statistical analysis. RC wrote the first draft of the manuscript. RC, CM, ES, JQ, JH, and YL wrote sections of the manuscript. All authors contributed to the manuscript revision, read, and approved the submitted version.

## Conflict of Interest

The authors declare that the research was conducted in the absence of any commercial or financial relationships that could be construed as a potential conflict of interest.

## Publisher’s Note

All claims expressed in this article are solely those of the authors and do not necessarily represent those of their affiliated organizations, or those of the publisher, the editors and the reviewers. Any product that may be evaluated in this article, or claim that may be made by its manufacturer, is not guaranteed or endorsed by the publisher.
