# Supplementary material for: Normative Data and Determinants of Macular, Disc, and Peripapillary Vascular Density in Healthy Myopic Children Using Optical Coherence Tomography Angiography
Source: Front Med (Lausanne). 2022 Jun 21;9:890294. doi: 10.3389/fmed.2022.890294 (PMC9253463; doi:10.3389/fmed.2022.890294)
Supplement: Supplementary file 1 [file Table_1.DOCX]

Supplementary Material

# Supplementary Tables

| Table 1. The relationships between possible factors and macular VD (**Pearson’s correlation, *R****)*. | | | | | | | | |
| --- | --- | --- | --- | --- | --- | --- | --- | --- |
| **Section** | **Gender** | **Age** | **AL** | **SER** | **SSI** | **CFT** | **CFV** | **eye** |
| **Superficial vascular density (SCP VD)** | | | | | | | | |
| Whole retina | 0.078* | -0.028 | -0.056 | 0.056 | 0.473** | -0.046 | -0.047 | .428** |
| Fovea | -0.190** | 0.035 | 0.198** | -0.066 | 0.021 | 0.042 | 0.042 | .867** |
| Parafovea | 0.046 | -0.024 | -0.044 | 0.054 | 0.411** | -0.071* | -0.073* | .332** |
| -Temporal | 0.081* | -0.004 | -0.032 | 0.014 | 0.327** | -0.044 | -0.045 | .275** |
| -Superior | 0.021 | -0.045 | -0.050 | 0.071* | 0.398** | -0.078* | -0.079* | .323** |
| -Nasal | 0.019 | -0.019 | -0.041 | 0.054 | 0.312** | -0.084* | -0.086* | .242** |
| -Inferior | -0.015 | -0.031 | 0.045 | 0.396** | -0.045 | -0.046 | -0.015 | .261** |
| Perifovea | 0.079* | 0.001 | -0.050 | 0.046 | 0.458** | -0.038 | -0.039 | .469** |
| -Temporal | 0.016 | -0.076* | -0.122** | 0.113** | 0.496** | -0.039 | -0.040 | .384** |
| -Superior | 0.138** | 0.017 | -0.085* | 0.038 | 0.380** | -0.045 | -0.046 | .472** |
| -Nasal | 0.070 | 0.080* | 0.068 | -0.037 | 0.299** | -0.035 | -0.036 | .395** |
| -Inferior | 0.058 | -0.004 | -0.020 | 0.037 | 0.421** | -0.018 | -0.019 | .439** |
| **Intermediate vascular density (ICP VD)** | | | | | | | |  |
| Whole retina | 0.134** | -0.035 | -0.139** | 0.091* | 0.581** | -0.036 | -0.037 | 0.131** |
| Fovea | -0.183** | 0.056 | 0.248** | -0.113** | 0.005 | 0.061 | 0.061 | 0.569** |
| Parafovea | 0.267** | -0.047 | -0.151** | 0.046 | 0.382** | -0.048 | -0.049 | 0.127** |
| -Temporal | 0.270** | -0.013 | -0.097** | 0.001 | 0.300** | -0.024 | -0.025 | 0.115** |
| -Superior | 0.222** | -0.059 | -0.159** | 0.075* | 0.396** | -0.079* | -0.080* | 0.130** |
| -Nasal | 0.259** | -0.016 | -0.112** | 0.001 | 0.297** | -0.047 | -0.048 | 0.118** |
| -Inferior | 0.231** | -0.075* | -0.174** | 0.081* | 0.390** | -0.024 | -0.024 | 0.119** |
| Perifovea | 0.125** | -0.038 | -0.131** | 0.090* | 0.576** | -0.042 | -0.043 | 0.128** |
| -Temporal | 0.143** | -0.045 | -0.140** | 0.089* | 0.504** | -0.027 | -0.028 | 0.114** |
| -Superior | 0.112** | 0.008 | -0.095** | 0.060 | 0.565** | -0.043 | -0.043 | 0.119** |
| -Nasal | 0.121** | -0.022 | -0.100** | 0.067 | 0.498** | -0.048 | -0.048 | 0.140** |
| -Inferior | 0.095* | -0.080* | -0.156** | 0.117** | 0.564** | -0.037 | -0.038 | 0.141** |
| **Deep vascular density (DCP VD)** | | | | | | | |  |
| Whole retina | -0.051 | 0.095** | 0.241** | -0.202** | -0.607** | 0.041 | 0.041 | 0.034 |
| Fovea | -0.160** | 0.136** | 0.355** | -0.246** | -0.438** | 0.082* | 0.082* | 0.490** |
| Parafovea | 0.014 | 0.113** | 0.191** | -0.198** | -0.560** | 0.032 | 0.032 | 0.023 |
| -Temporal | 0.037 | 0.116** | 0.176** | -0.195** | -0.551** | 0.044 | 0.044 | 0.016 |
| -Superior | 0.021 | 0.081* | 0.155** | -0.162** | -0.471** | 0.001 | 0.001 | -0.006 |
| -Nasal | -0.002 | 0.103** | 0.195** | -0.195** | -0.523** | 0.038 | 0.038 | 0.057 |
| -Inferior | 0.001 | 0.120** | 0.190** | -0.188** | -0.553** | 0.034 | 0.034 | 0.036 |
| Perifovea | -0.056 | 0.103** | 0.240** | -0.203** | -0.620** | 0.037 | 0.037 | 0.047 |
| -Temporal | -0.021 | 0.139** | 0.228** | -0.211** | -0.581** | 0.030 | 0.030 | 0.047 |
| -Superior | -0.074 | 0.027 | 0.189** | -0.135** | -0.548** | 0.026 | 0.026 | 0.034 |
| -Nasal | -0.048 | 0.082* | 0.229** | -0.196** | -0.567** | 0.054 | 0.054 | 0.056 |
| -Inferior | -0.060 | 0.137** | 0.249** | -0.216** | -0.619** | 0.028 | 0.028 | 0.071* |
| **Choriocapillaris vascular density (CC VD)** | | | | | | | | |
| Whole retina | -0.053 | 0.171** | 0.234** | -0.180** | -0.161** | 0.060 | 0.060 | 0.458** |
| Fovea | -0.042 | -0.006 | 0.076* | -0.061 | 0.145** | 0.021 | 0.022 | 0.382** |
| Parafovea | -0.009 | 0.140** | 0.216** | -0.167** | -0.180** | 0.023 | 0.023 | 0.517** |
| -Temporal | -0.042 | 0.123** | 0.197** | -0.131** | -0.196** | 0.017 | 0.017 | 0.431** |
| -Superior | 0.034 | 0.146** | 0.230** | -0.212** | -0.143** | 0.029 | 0.030 | 0.483** |
| -Nasal | -0.017 | 0.131** | 0.179** | -0.133** | -0.170** | 0.041 | 0.041 | 0.476** |
| -Inferior | -0.012 | 0.101** | 0.167** | -0.122** | -0.136** | -0.005 | -0.004 | 0.498** |
| Perifovea | -0.057 | 0.198** | 0.264** | -0.205** | -0.231** | 0.063 | 0.063 | 0.450** |
| -Temporal | -0.055 | 0.189** | 0.279** | -0.197** | -0.247** | 0.054 | 0.054 | 0.341** |
| -Superior | -0.052 | 0.208** | 0.296** | -0.271** | -0.244** | 0.070 | 0.069 | 0.418** |
| -Nasal | -0.027 | 0.154** | 0.129** | -0.109** | -0.189** | 0.067 | 0.068 | 0.415** |
| -Inferior | -0.068 | 0.159** | 0.242** | -0.159** | -0.155** | 0.037 | 0.037 | 0.436** |
| **FAZ and FD-300** | | | | | | | | |
| FAZ area | 0.194** | -0.036 | -0.014* | 0.022 | 0.047 | 0.014 | 0.015 | 0.538** |
| FAZ perimeter | 0.197** | -0.042 | -0.045 | 0.037 | 0.061 | 0.006 | 0.007 | 0.416** |
| FD-300 area | 0.111** | -0.039 | -0.097** | 0.047 | 0.277** | -0.067 | -0.067 | 0.144** |
| FD-300 length | 0.147** | -0.056 | -0.065 | 0.066 | 0.277** | -0.010 | -0.009 | 0.079* |

Gender 1: boy, 2: girl; Eye 1: right, 2: left; AL: axial length; SER: spherical equivalent refraction; SSI: signal strength index; CFT: central foveal thickness; CFV: central foveal volume. **P*＜0.05 and * **P*＜0.01 were marked as red color.

| Table 2. The relationships between possible factors and disc and peripapillary vascular density (**Pearson’s correlation, R**). | | | | | | | | |
| --- | --- | --- | --- | --- | --- | --- | --- | --- |
| **Section** | **Gender** | **Age** | **AL** | **SER** | **SSI** | **Disc area** | **TS** | **Eye** |
| Whole | 0.171** | -0.126** | -0.253** | 0.191** | 0.048 | 0.197** | 0.077* | 0.471** |
| Whole capillary | 0.124** | -0.062 | -0.150** | 0.102** | -0.021 | 0.166** | 0.076* | 0.412** |
| Inside disc | 0.105** | 0.046 | 0.040 | -0.051 | 0.082* | 0.087* | 0.020 | 0.392** |
| Inside disc capillary | 0.074 | 0.180** | 0.218** | -0.224** | 0.038 | 0.142** | 0.054 | 0.492** |
| Peripapillary | 0.144** | -0.067 | -0.182** | 0.130** | -0.052 | 0.210** | 0.073* | 0.471** |
| Peripapillary capillary | 0.096* | -0.004 | -0.083* | 0.051 | -0.121** | 0.142** | 0.052 | 0.418** |
| -Nasal superior | 0.076* | -0.027 | -0.041 | 0.045 | 0.022 | 0.138** | -0.044 | 0.364** |
| -Nasal inferior | 0.028 | -0.012 | -0.057 | 0.092* | 0.034 | 0.221** | -0.023 | 0.404** |
| -Inferior nasal | 0.091* | -0.026 | -0.162** | 0.093** | -0.128** | 0.069 | -0.008 | 0.035 |
| -Inferior temporal | 0.094* | 0.014 | -0.041 | 0.012 | -.0187** | 0.001 | 0.022 | 0.279** |
| -Temporal superior | -0.020 | 0.087* | 0.072* | -0.090* | -0.149** | 0.000 | 0.220** | 0.084* |
| -Temporal inferior | 0.070 | 0.071* | 0.019 | -0.067 | -0.130** | 0.047 | 0.340** | 0.120** |
| -Superior temporal | 0.045 | -0.016 | -0.056 | 0.008 | -0.091* | 0.102** | -0.030 | 0.317** |
| -Superior nasal | 0.105** | -0.059 | -0.086* | 0.075* | -0.098** | 0.029 | -0.087* | 0.369** |

AL: axial length; SER: spherical equivalent refraction; SSI: signal strength index; CFT: central foveal thickness; CFV: central foveal volume. TS, total thickness of never fiber. **P*＜0.05 and * **P*＜0.01 were marked as red color.
